# Longitudinal association between perceived stress and depressive symptoms among student-athletes: the mediating roles of positive reframing and behavioral disengagement and moderating role of stress mindset

**DOI:** 10.3389/fpsyg.2026.1938720

**Published:** 2026-09-08

**Authors:** Ziyou Huang, Lei Pan, Yajie Xu, Xiaofen Li, Weida Deng

**Affiliations:** 1 School of Sports and Health, Anqing Normal University, Anqing, China; 2 School of Art, Beijing Sport University, Beijing, China; 3 Center for Teaching and Learning Development, Xiamen University, Xiamen, China

**Keywords:** behavioral disengagement, depressive symptoms, perceived stress, positive reframing, stress mindset, student-athletes

## Abstract

**Introduction:**

Student-athletes are exposed to multiple academic and athletic demands, which increase their vulnerability to depressive symptoms. This study examined the longitudinal association between perceived stress and depressive symptoms among Chinese student-athletes. We also focused on the mediating roles of positive reframing and behavioral disengagement and the moderating role of stress mindset.

**Methods:**

Of the 840 participants assessed at baseline, 769 (retention rate = 91.5%; 64.1% male; Mage = 20.3 years) completed all three waves, which were conducted at 6-month intervals. Structural equation modeling was used to test a moderated mediation model while controlling for autoregressive effects and relevant covariates.

**Results:**

The longitudinal mediation model indicated that higher perceived stress at T1 was positively associated with depressive symptoms at T3 (β = 0.20, 95% CI = [0.12, 0.28]). Significant indirect associations were observed through lower positive reframing (β = 0.018, 95% CI [0.006, 0.030]) and higher behavioral disengagement at T2 (β = 0.022, 95% CI [0.006, 0.038]). The moderated mediation model found that stress mindset moderated the associations of perceived stress with positive reframing (β = 0.06, 95% CI [0.02, 0.10]), behavioral disengagement (β = −0.08, 95% CI [−0.13, −0.03]), and depressive symptoms (β = −0.08, 95% CI [−0.13, −0.03]), with weaker associations at higher levels of stress mindset.

**Conclusions:**

Positive reframing and behavioral disengagement were prospectively associated with the link between perceived stress and later depressive symptoms, while stress mindset was associated with weaker stress-related associations. Given the observational design, these findings do not establish causal relationships but provide longitudinal evidence on coping and stress-related beliefs in student-athletes.

## 1 Introduction

Student-athletes represent a unique population that is simultaneously exposed to academic demands and intensive athletic training, creating a distinctive combination of academic and sport-related pressures (Zhang and Wang, 2025). Although comparative studies have not consistently shown higher depressive symptoms among student-athletes than among non-athlete students, depressive symptoms remain common and clinically relevant in collegiate athletic populations (Armstrong and Oomen-Early, 2009; Usenik and Kranjec, 2025; Woodson et al., 2025). Student-athletes should also be distinguished from elite athletes, who may not simultaneously face academic demands. Epidemiological studies indicate that a substantial proportion of elite athletes experience depressive symptoms (Gouttebarge et al., 2019; Rice et al., 2019; Runacres and Marshall, 2024), with recent meta-analytic evidence reporting a pooled prevalence of 30.7% among female athletes (Beisecker et al., 2024). These findings highlight depressive symptoms as a significant mental health concern in competitive sport contexts. Importantly, depressive symptoms among student-athletes are associated with a range of adverse outcomes, including impaired performance, burnout, greater injury risk, and an increased likelihood of sport dropout (Dišlere et al., 2025; Gil-Caselles et al., 2024; McCluskey et al., 2025). Persistent depressive symptoms are linked to poor long-term well-being and hinder athletes' academic and athletic development (Rocco et al., 2025). Despite growing awareness of mental health in sport, depressive symptoms remain an important concern in student-athlete populations.

Within the stress–health literature, perceived stress has been identified as an important predictor of depressive symptoms. Perceived stress refers to individuals' subjective evaluations of the extent to which life circumstances are experienced as unpredictable, uncontrollable, and overwhelming (Lee, 2012). According to stress and coping theory, psychological outcomes are shaped by exposure to stressors, individuals' subjective appraisals of those stressors, and the coping resources available to them (Folkman, 2013). Consistent with this theory, empirical studies across diverse populations, including students, healthcare workers, and athletes, have documented a positive association between perceived stress and depressive symptoms (Heinen et al., 2017; Jochmann et al., 2024; Pänkäläinen et al., 2024; Racic et al., 2017). In athletic contexts, perceived stress arises from multiple sources, such as training load, performance expectations, injury concerns, academic pressures, and conflicts between athletic and educational roles (Nixdorf et al., 2020; Nuetzel, 2025). When these stressors accumulate, they gradually increase student-athletes' vulnerability to depressive symptoms. However, much of the prior research has relied on cross-sectional designs, which limits understanding of the temporal relationship between perceived stress and depressive symptoms. Additionally, relatively few studies have examined factors that could buffer or exacerbate this association in athletic populations. Consequently, it remains unclear how perceived stress is associated with later depressive symptoms and under what conditions this association becomes stronger or weaker among student-athletes.

Stress and coping theory further emphasizes the role of coping strategies in shaping individuals' psychological responses to stress (Folkman, 2013). Coping refers to the cognitive and behavioral efforts individuals use to manage demands appraised as stressful (Spătaru et al., 2024). However, coping is a multidimensional construct. Different coping strategies work through distinct psychological pathways (Stanisławski, 2019; Vannucci et al., 2018). Thus, it is important to examine specific strategies that are theoretically related to the stress experiences of student-athletes (Kroemeke, 2019). In the present study, we focused on positive reframing and behavioral disengagement because these two strategies capture two contrasting yet highly meaningful responses to stress, i.e., constructive cognitive reinterpretation and maladaptive behavioral withdrawal (Faulk et al., 2013). This selection was not intended to represent the full coping repertoire or to reduce coping to a simple adaptive-maladaptive dichotomy. Rather, these two strategies were selected because they correspond to two targeted pathways that are especially relevant to student-athletes: the cognitive reinterpretation of stress and withdrawal from effortful coping. Other coping strategies, such as problem-focused coping, acceptance, and support seeking, may also be important, but they reflect additional mechanisms that were beyond the focused model tested in this study.

Positive reframing was chosen because it is conceptually well aligned with the appraisal-centered perspective of stress and coping theory. Perceived stress reflects individuals' subjective evaluations of situations as overwhelming, uncontrollable, or unpredictable (Michaelides et al., 2016), and positive reframing directly targets this evaluative process by enabling individuals to reinterpret stressful experiences in a more constructive or manageable way (Chaaya et al., 2025). Positive reframing changes the meaning attached to stressors, which helps to reduce emotional burden and preserve psychological adjustment (Robbins et al., 2019; Strauss et al., 2016). This strategy is meaningful for student-athletes. This population often faces stressors, such as performance pressure, injury concerns, team selection, academic demands, and role conflict between sport and education, all of which cannot be immediately removed (Stambulova and Wylleman, 2019). For such persistent or difficult-to-control stressors, cognitive reappraisal may be relevant because it allows athletes to adjust the meaning of the stressor even when the stressor itself remains present (Troy et al., 2018). As a result, the ability to cognitively reframe adversity represents an important protective resource against depressive symptoms under these conditions (Compas et al., 2017; Robbins et al., 2019). Prior research has similarly linked adaptive coping strategies, i.e., positive reframing, to lower levels of depressive symptoms and better psychological adjustment (Rahmani et al., 2024; Rodrigues et al., 2023; Song et al., 2021).

Behavioral disengagement was selected because it represents a particularly detrimental form of maladaptive coping that is highly relevant in high-demand performance environments. Behavioral disengagement refers to reducing effort, withdrawing from goal-directed action, and giving up attempts to manage stressors (Pilecka et al., 2025). It reflects a breakdown in active self-regulation when individuals perceive demands as exceeding their coping ability. Such withdrawal is consequential among student-athletes because success in both sport and academics depends heavily on sustained effort, persistence, and self-discipline (Linnér et al., 2025). When stressed student-athletes begin to behaviorally disengage, they easily fail to address stressors effectively, which are then related to depressive symptoms (Compas et al., 2017). Therefore, compared with broader maladaptive coping categories, behavioral disengagement provides a more precise indicator of the withdrawal process through which stress is linked to depressive symptoms (Feinstein et al., 2017). This pathway is theoretically distinct from other coping responses because it captures reduced persistence and disengagement from goal-directed action, rather than the absence of coping in general.

Importantly, positive reframing and behavioral disengagement were examined together because they reflect two complementary coping mechanisms through which perceived stress may be linked to depressive symptoms. Positive reframing represents a potentially protective pathway, through which individuals cognitively regulate the meaning of stress and reduce its emotional impact (Troy et al., 2018; Zhan et al., 2017). In contrast, behavioral disengagement represents a risk pathway, through which stress is connected with withdrawal, helplessness, and reduced coping effort (Compas et al., 2017). Examining these two strategies simultaneously allows for a deeper understanding of how perceived stress is linked to depressive symptoms among student-athletes. Thus, the model was designed to test two theoretically central but selective coping pathways, rather than to provide an exhaustive account of all possible coping mechanisms.

Based on stress and coping theory (Folkman, 2013), these strategies may be key mediating mechanisms linking perceived stress to depressive symptoms. High levels of perceived stress reduce individuals' ability to engage in constructive cognitive coping, e.g., positive reframing, while increasing the likelihood of relying on withdrawal-based responses, e.g., behavioral disengagement (Foster et al., 2023; She et al., 2021). In turn, lower positive reframing and greater behavioral disengagement are associated with vulnerability to depressive symptoms. Although previous studies have shown that coping mediates the relationship between perceived stress and mental health outcomes (Huda et al., 2021; Klainin-Yobas et al., 2014; Wang and Wang, 2019), several limitations remain. Much of this literature has focused on broad coping dimensions rather than specific coping strategies. Moreover, little is known about whether positive reframing and behavioral disengagement simultaneously mediate the longitudinal association between perceived stress and depressive symptoms in student-athletes. Consequently, focusing on positive reframing and behavioral disengagement allows us to examine two specific psychological pathways through which perceived stress is prospectively associated with depressive symptoms over time in this population.

Stress mindset has recently gained increasing attention as a potential factor that shapes the impact of stress on depressive symptoms. It refers to individuals' overarching beliefs about whether stress is beneficial or harmful (Crum et al., 2013). Specifically, a stress-is-enhancing mindset reflects the belief that stress can facilitate growth, learning, and performance, whereas a stress-is-debilitating mindset emphasizes its damaging consequences (Horiuchi et al., 2024; Williams and Ginty, 2024). According to stress and coping theory, these beliefs influence how stressful experiences are interpreted and how individuals respond when facing stress (Mansell and Turner, 2023). Accordingly, individuals who perceive stress as potentially enhancing may be better to sustain adaptive responses and avoid maladaptive reactions under stressful conditions (Xiang et al., 2025), thereby lowering their risk of depressive symptoms.

Empirical findings support this view. For example, among college students, the positive association between perceived stress and depressive symptoms is stronger for those endorsing a stress-is-debilitating mindset than those holding a stress-is-enhancing mindset (Huebschmann and Sheets, 2020). Recent evidence also indicates that stress mindset shapes the link between daily life stress and emotional well-being, with a stress-is-enhancing mindset buffering increases in negative emotions and reducing declines in well-being over time (Zeng et al., 2024). However, previous studies have generally examined coping as a mediator or stress mindset as a moderator separately. It therefore remains unclear whether stress mindset also conditions the specific coping pathways linking perceived stress to later depressive symptoms. By examining positive reframing and behavioral disengagement as parallel mediators and stress mindset as a moderator within the same longitudinal model, the present study addresses two related questions: through which coping responses perceived stress is prospectively associated with depressive symptoms, and whether the strength of these pathways varies according to stress-related beliefs.

Building on the prior literature, the present study tested the longitudinal association between perceived stress and depressive symptoms among Chinese student-athletes, the mediating roles of positive reframing and behavioral disengagement, and the moderating role of stress mindset. By examining positive reframing and behavioral disengagement as parallel mediators and stress mindset as a moderator within the same three-wave longitudinal model, the present study extends prior stress-coping research in two ways. First, it distinguishes between two theoretically contrasting coping pathways: constructive cognitive reinterpretation and withdrawal-based disengagement. Second, it examines whether stress mindset moderates these coping pathways, rather than only the direct association between perceived stress and depressive symptoms. Thus, the study clarifies both how perceived stress is prospectively associated with later depressive symptoms and for whom these associations may be stronger or weaker. Thus, we had the following hypotheses:

(H1) Higher perceived stress would be associated with lower positive reframing, which in turn would be associated with greater depressive symptoms, resulting in a positive indirect association between perceived stress and depressive symptoms.

(H2) Higher perceived stress would be associated with greater behavioral disengagement, which in turn would be associated with greater depressive symptoms, also resulting in a positive indirect association.

(H3) Stress mindset would moderate the longitudinal associations between perceived stress and these coping strategies, such that a stronger stress-is-enhancing mindset would weaken the negative association between perceived stress and positive reframing and attenuate the positive association between perceived stress and behavioral disengagement.

(H4) Stress mindset would also moderate the direct association between perceived stress and depressive symptoms, such that the positive association between perceived stress and depressive symptoms would be weaker among individuals with a stronger stress-is-enhancing mindset.

## 2 Methods

### 2.1 Participants and data collection

This study employed a three-wave longitudinal design. Baseline data were collected in February 2025 (Time 1), followed by two additional waves at 6-month intervals in August 2025 (Time 2) and February 2026 (Time 3). Participants were recruited from sports schools and university athletic teams in Anhui Province, China. A convenience sampling approach was used. The research team contacted four institutions, including two sports schools and two universities with athletic teams, and invited eligible student-athletes from 20 teams to participate. Recruitment and baseline assessment were conducted between February 3 and February 25, 2025. Eligible participants were student-athletes who met the following criteria: (a) aged 18 years or above; (b) currently enrolled as full-time students in a sports school or university; and (c) formally registered in a school- or university-based athletic team or training group. Most participants were preparing for or participating in school-level, provincial, or national competitions during the academic year. Athletes were excluded if they were younger than 18 years, were not currently enrolled as students, were not engaged in organized athletic training, had withdrawn from regular training because of injury or other reasons, or were unable to provide informed consent. A total of 862 athletes met the inclusion criteria and were invited to participate. Of these, 840 completed the baseline survey, yielding a baseline response rate of 97.4%. Reasons for non-participation included schedule conflicts, absence during data collection, and unwillingness to provide informed consent. At each wave, data were collected using a structured, self-administered online questionnaire. Trained research staff collaborated with coaches and administrative personnel to distribute the survey link or QR code to eligible participants and to coordinate data collection. The online questionnaire required responses to all items before submission, so submitted questionnaires did not contain item-level missing data.

To match participants' responses across the three waves, participants were asked to provide their mobile phone numbers. These phone numbers were used solely for longitudinal matching purposes and were kept strictly confidential. To protect privacy, identifying information was stored separately from questionnaire responses and replaced with anonymous participant IDs after linkage. The linkage file was stored in password-protected electronic files and was accessible only to the investigators. Mobile phone numbers were not included in the analytic dataset and were not used in any statistical analyses. After longitudinal matching and data verification were completed, the linkage file containing mobile phone numbers was deleted. All identifying information was securely stored and accessible only to the research team, and was not used for any purpose other than data matching.

This study was conducted in accordance with the Declaration of Helsinki. Prior to participation, all athletes received an explanation of the study's purpose, procedures, and confidentiality measures. Written informed consent was obtained from all participants. This study was approved by the Research Ethics Committee of Anqing Normal University. (Approval number: AQNU2025211). Because the PHQ-9 includes an item assessing thoughts of self-harm, a safety protocol was implemented during data collection. Participants were informed that they could discontinue participation at any time if they felt uncomfortable. If a participant endorsed self-harm thoughts or showed obvious distress, trained research staff would speak with the participant privately, provide information about school or university counseling services, and recommend timely professional support.

**Figure 1:**
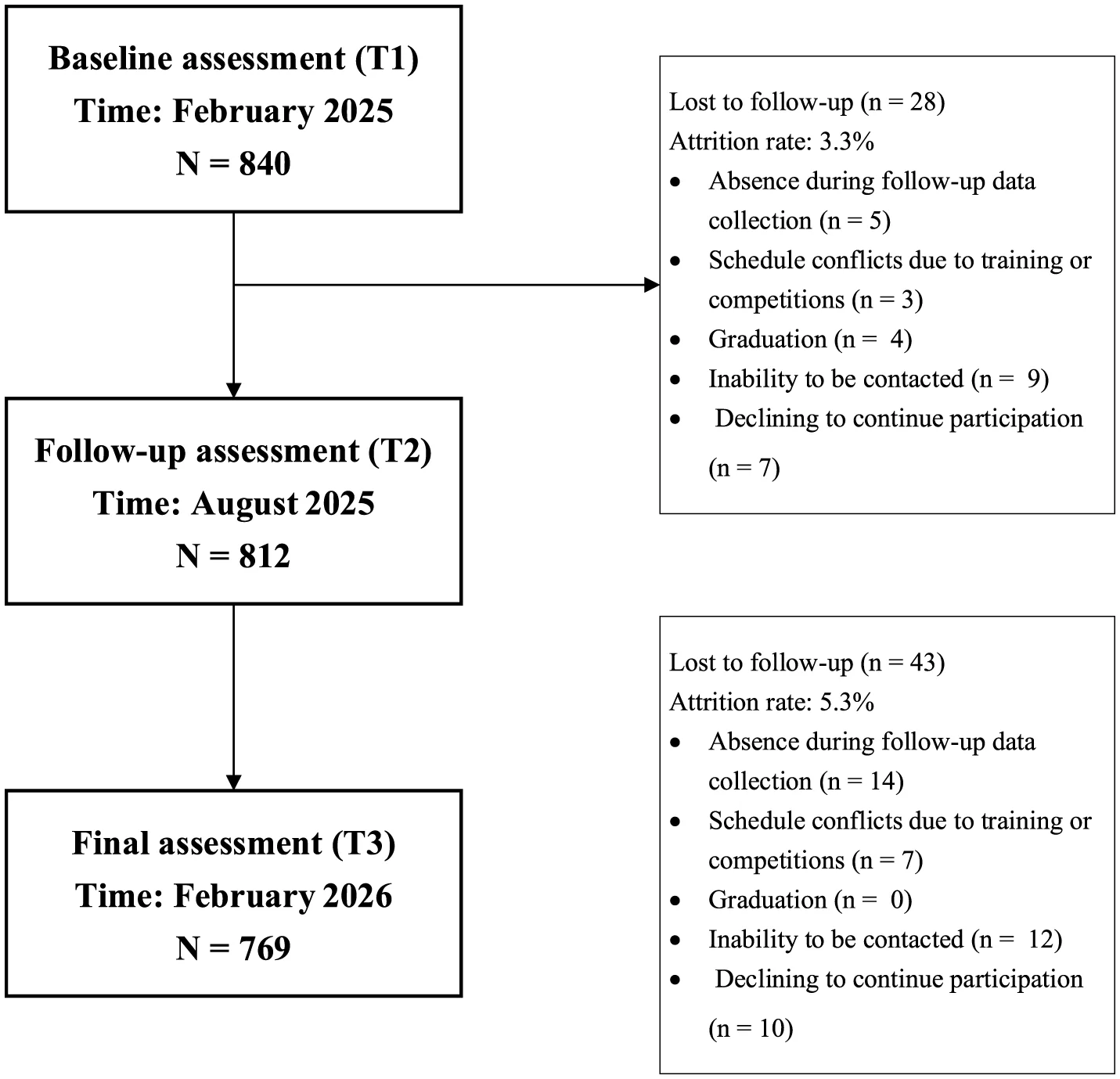
Participants flowchart.

At baseline (T1), 840 participants completed the survey. At T2, 812 participants completed the follow-up survey. At T3, 769 participants completed the final survey and had valid matched data across all three waves, corresponding to a three-wave retention rate of 91.5%. Reasons for attrition included absence during follow-up data collection, schedule conflicts due to training or competitions, graduation, inability to be contacted, and declining to continue participation. The participant flowchart is presented in Figure 1.

### 2.2 Instruments

#### 2.2.1 Demographic variables

Age, sex, perceived family financial situation, type of sport, years of training, and weekly training hours were collected.

#### 2.2.2 Depressive symptoms

Depressive symptoms were assessed using the 9-item Patient Health Questionnaire (Kroenke et al., 2001). It asks respondents to report how often they experienced depressive symptoms during the previous 14 days. The instrument has nine items, which are rated on a four-point scale ranging (not at all = 0 to nearly every day = 3), with higher scores showing more severe symptoms. It has been used in Chinese population (Lu et al., 2024). In the present study, the Cronbach's α of depressive symptoms at T1, T2, T3 was 0.82, 0.84, and 0.86, respectively.

#### 2.2.3 Positive reframing

Positive reframing was assessed using the positive reframing subscale of the Brief COPE (Carver, 1997). It has two items that assess the extent to which individuals reinterpret stressful situations in a more constructive manner. Participants rated on a four-point scale ranging (I haven't been doing this at all = 1 to I've been doing this a lot = 4). Higher scores show greater use of positive reframing. The Brief COPE has been examined in Chinese samples and has been used in research with Chinese athletes (Su et al., 2015). Because this subscale contains only two items, reliability was evaluated using inter-item correlations and Spearman-Brown coefficients (Eisinga et al., 2013). In this study, the inter-item correlations for positive reframing were 0.70 at T1 and 0.75 at T2, and the Spearman-Brown coefficients were 0.82 and 0.86, respectively. The Cronbach's α for positive reframing at T1 and T2 was 0.82 and 0.86, respectively.

#### 2.2.4 Behavioral disengagement

Behavioral disengagement was measured using the behavioral disengagement subscales of the Brief COPE (Carver, 1997). It has two items that measure the extent to which individuals withdraw effort and give up attempts to deal with stressful situations. Participants rated on a four-point scale (I haven't been doing this at all = 1 to I've been doing this a lot = 4). Higher scores suggest greater use of behavioral disengagement. It has been used in Chinese university students and shown acceptable psychometrics (Lu et al., 2024). The reliability was evaluated using inter-item correlations and Spearman-Brown coefficients (Eisinga et al., 2013). In this study, the inter-item correlations for behavioral disengagement were 0.74 at T1 and 0.82 at T2, and the Spearman-Brown coefficients were 0.85 and 0.90, respectively. In this study, the Cronbach's α for behavioral disengagement at T1 and T2 was 0.85 and 0.90, respectively.

#### 2.2.5 Perceived stress

Perceived stress was measured using the 10-item Perceived Stress Scale (Taylor, 2015). Participants rated each item on a five-point scale (never = 0 to very often = 4). The Chinese PSS-10 has shown acceptable reliability and validity in Chinese university students (Lu et al., 2017). In the present study, the Cronbach's α for perceived stress at T1 was 0.85.

#### 2.2.6 Stress mindset

Stress mindset was measured using the 8-item Stress Mindset Measure-General (SMM-G; Crum et al., 2013). Responses were rated on a five-point Likert scale (strongly disagree = 0 to strongly agree = 4). The scale includes four stress-is-enhancing items and four stress-is-debilitating items. Items 2, 4, 6, and 8, which reflect a stress-is-debilitating mindset, were reverse-coded before computing the total score. Total scores range from 0 to 32, with higher scores indicating a stronger stress-is-enhancing mindset. The Chinese version of the Stress Mindset Measure-General (SMM-G) has been used in Chinese first-year college students and showed acceptable internal consistency (Zhao et al., 2023). In the present study, the Cronbach's α for stress mindset at T1 was 0.81.

### 2.3 Data analyses

#### 2.3.1 Preliminary analyses

Attrition analyses were conducted to examine whether participants retained across all three waves differed from those lost to follow-up. Independent-samples t-tests were used for continuous variables, and chi-square tests were used for categorical variables. Descriptive statistics were first calculated for all study variables. The associations between perceived stress at T1, stress mindset at T1, positive reframing at T2, behavioral disengagement at T2, and depressive symptoms at T3 were tested using Pearson correlation.

Before testing the structural model, the T1 PSS-10 measurement model was examined using confirmatory factor analysis. Model fit indices, standardized factor loadings, and residual variances of the latent perceived-stress construct were evaluated.

#### 2.3.2 Longitudinal mediation model

Structural equation modeling (SEM) was conducted to examine the longitudinal model. Perceived stress at T1 was the predictor, positive reframing at T2 and behavioral disengagement at T2 were parallel mediators, and depressive symptoms at T3 was the outcome variable. To strengthen the temporal ordering of the model, autoregressive effects were controlled for, including positive reframing at T1 predicting positive reframing at T2, behavioral disengagement at T1 predicting behavioral disengagement at T2, and depressive symptoms at T2 predicting depressive symptoms at T3. Depressive symptoms at T2 were specified as the autoregressive predictor of depressive symptoms at T3 because T2 was the most proximal prior assessment of the outcome. This specification follows the logic of first-order autoregressive longitudinal modeling and longitudinal mediation analysis, in which adjacent-wave stability paths are commonly used to account for continuity in the same construct over time (Jose, 2016; Little, 2013). Similar adjacent-wave autoregressive controls have also been used in recent longitudinal mediation research (Shao et al., 2026). This specification means that the paths to T3 depressive symptoms should be interpreted as associations with later depressive symptoms after accounting for proximal symptom stability, rather than as total effects on depressive symptoms across the full study period.

Because this was a longitudinal cohort study, all eligible student-athletes from the participating institutions and teams were invited to participate. The final analytic sample included 769 participants with matched data across all three waves. This sample size was considered adequate for the planned SEM because SEM sample-size requirements depend on model complexity, missing data, indicator reliability, and expected path sizes rather than on a single universal rule (Muthén and Muthén, 2002; Wolf et al., 2013). The sample also provided an acceptable observations-to-parameters ratio according to the N:q framework (Jackson, 2003; Kline, 2016). In addition, the model controlled for age, sex, perceived family financial situation, years of training, and weekly training hours. Age and years of training were entered as continuous covariates; sex was coded as 1 = male and 2 = female; perceived family financial situation and weekly training hours were entered as ordered numeric covariates. Perceived stress was modeled as a single latent factor indicated by the PSS-10 items because it was the focal predictor in the model and item-level latent modeling allowed measurement error in perceived stress to be accounted for. This factor was interpreted as representing general perceived stress. Positive reframing and behavioral disengagement were represented by observed subscale scores because each Brief COPE subscale contained only two items. Depressive symptoms and stress mindset were represented by observed total scores to maintain model parsimony and because both scales showed acceptable internal consistency in the present sample.

#### 2.3.3 Moderated mediation model

To further test whether stress mindset moderated the pathways, a moderated mediation model was estimated using SEM. Perceived stress at T1 was represented as a latent variable indicated by the 10 PSS-10 items, whereas stress mindset at T1 was represented by the observed total score. Stress mindset was grand-mean centered before estimating the interaction. The interaction between latent perceived stress and observed stress mindset was estimated using the XWITH command in Mplus (version 8.3; Muthén & Muthén, Los Angeles, CA, USA). Because XWITH requires numerical integration, the moderated mediation model was estimated using robust maximum likelihood estimation (MLR) with TYPE = RANDOM and ALGORITHM = INTEGRATION. Conditional effects at low (−1 SD) and high (+1 SD) levels of stress mindset were estimated using MODEL CONSTRAINT.

Distributional diagnostics were examined for the depressive-symptom total scores. Depressive symptoms showed positive but non-severe skewness and kurtosis at T2 (skewness = 1.79, kurtosis = 3.80) and T3 (skewness = 1.19, kurtosis = 2.88). These values were below commonly used thresholds for severe non-normality in SEM (Kline, 2016). In the SEM, depressive symptoms were represented by observed total scores rather than ordinal item-level indicators. Therefore, maximum-likelihood estimation was retained. Indirect effects in the longitudinal mediation model were examined using bias-corrected bootstrap 95% confidence intervals based on 5,000 resamples. Conditional effects in the moderated mediation model were estimated using MODEL CONSTRAINT. Because the online questionnaire required responses to all items before submission, there was no item-level missingness among submitted questionnaires. The SEM analyses were conducted using complete cases with valid matched data across all three waves (*n* = 769).

Model fit was evaluated using multiple indices: Comparative Fit Index (CFI) and Tucker–Lewis Index (TLI) values of 0.90 or higher, and Root Mean Square Error of Approximation (RMSEA) and Standardized Root Mean Square Residual (SRMR) values of 0.08 or lower (Kline, 2016). SEM analyses were conducted in Mplus version 8.3. The other analyses were derived in IBM SPSS Statistics (version 26.0; IBM Corp., Armonk, NY, USA). The two-tailed *p*-value of less than 0.05 is statistical significance. This study reported the Standardized path coefficients (β) were reported for this study.

The study and analyses were not preregistered. Accordingly, the hypotheses were theory-driven, while follow-up probing of conditional indirect effects was treated as exploratory.

## 3 Results

### 3.1 Results of attrition analyses

Attrition analyses showed no significant differences between participants who completed all three waves and those lost to follow-up in terms of sex, perceived family financial situation, type of sport, years of training, and weekly training hours, age, perceived stress, stress mindset, positive reframing, behavioral disengagement, and depressive symptoms (Supplementary Table 1). The results suggest that attrition was unlikely to substantially bias the main findings.

### 3.2 Descriptive analysis

The characteristics of participants who completed all three waves are presented in Table 1. The mean age was 20.3-year-old (SD = 2.4), and the average years of training was 13.2 (SD = 2.1). 64.1% were males and 35.9% were females. 23.9% reported below average family financial situation. In terms of sport type, participants were involved in team sports (47.7%), individual sports (23.4%), weight-class sports (11.2%), and aesthetic sports (17.7%). 62.6% trained more than 16 h per week.

**Table 1:** Characteristics of participants who completed all three waves of the survey.

Continuous variable	Mean	SD
Age	20.3	2.4
Years of training	13.2	2.1
Categorical variable	*n*	%
**Sex**	
Male	493	64.1
Female	276	35.9
**Perceived family financial situation**	
Very poor/poor	184	23.9
Average	539	70.1
Good/very good	46	6.0
**Type of sport**	
Team sports	367	47.7
Individual sports	180	23.4
Weight-class sports	86	11.2
Aesthetic sports	136	17.7
**Weekly training hours**	
< 5 h	75	9.8
5–10 h	61	7.9
11–15 h	152	19.8
16–20 h	239	31.1
>20 h	242	31.5

As shown in Table 2, the mean (SD) score for perceived stress at T1, stress mindset at T1, positive reframing at T2, behavioral disengagement at T2, and depressive symptoms at T3 was 14.8 (6.3), 18.7 (4.3), 6.9 (1.3), 3.7 (1.4), and 5.0 (3.9), respectively.

**Table 2:** Mean, SD, and correlations.

Variable	1	2	3	4	5
1. Perceived stress at T1	1				
2. Stress mindset at T1	−0.28^***^	1			
3. Positive reframing at T2	−0.30^***^	0.26^***^	1		
4. Behavioral disengagement at T2	0.26^***^	−0.20^***^	−0.28^***^	1	
5. Depressive symptoms at T3	0.54^***^	−0.19^***^	−0.29^***^	0.19^***^	1
Mean	14.8	18.7	6.9	3.7	5.0
SD	6.3	4.3	1.3	1.4	3.9

^***^p < 0.001.

### 3.3 Correlations

As presented in Table 2, perceived stress at T1 was negatively correlated with stress mindset at T1 (*r* = −0.28) and positive reframing at T2 (*r* = −0.30), but positively correlated with behavioral disengagement at T2 (*r* = 0.26) and depressive symptoms at T3 (*r* = 0.54). Stress mindset at T1 was positively associated with positive reframing at T2 (*r* = 0.26) and negatively associated with behavioral disengagement at T2 (*r* = −0.20) and depressive symptoms at T3 (*r* = −0.19). Positive reframing at T2 was negatively correlated with behavioral disengagement at T2 (*r* = −0.28) and depressive symptoms at T3 (*r* = −0.29), whereas behavioral disengagement at T2 was positively associated with depressive symptoms at T3 (*r* = 0.19).

### 3.4 Measurement model of perceived stress

The T1 PSS-10 measurement model showed good fit, χ^2^(*35*) = 94.93, CFI = 0.946, TLI = 0.935, RMSEA = 0.054, 95% CI [0.043, 0.065], SRMR = 0.042. As shown in Supplementary Table 2, standardized factor loadings ranged from 0.51 to 0.66, and residual variances ranged from 0.57 to 0.74. These results supported the use of perceived stress as a latent construct in the mediation component of the SEM.

### 3.5 Longitudinal mediation model

**Figure 2:**
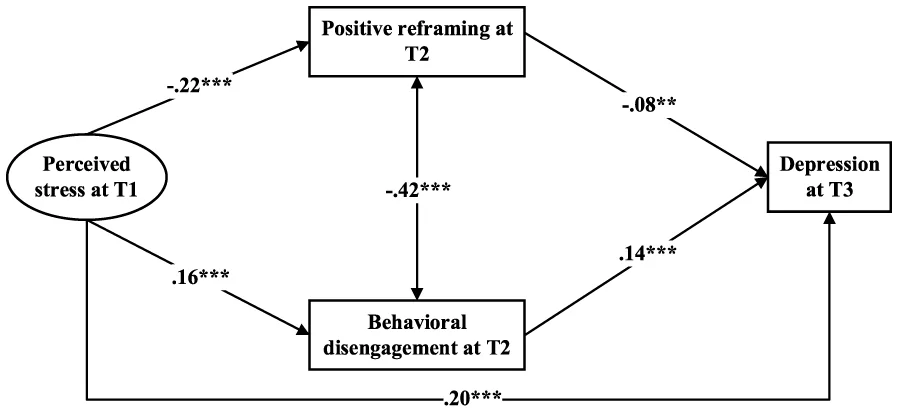
Structural equation modeling of the mediation effect of positive reframing and behavioral disengagement at T2 between perceived stress at T1 and depression at T3. *, *p* < 0.05, **, *p* < 0.01, ***, *p* < 0.001.

The results of the SEM are presented in Figure 2. The SEM yielded satisfactory model fit indices ($\chi_{\left(18\right)}^{2}$ = 49.44, *p* < 0.001, CFI = 0.979, TLI = 0.965, SRMR = 0.022, RMSEA = 0.034, 95% CI = [0.026, 0.042]).

As shown in Table 3, perceived stress at T1 significantly predicted both mediators at T2. Specifically, perceived stress was linked to positive reframing negatively (β = −0.22, SE = 0.04, 95% CI = [−0.30, −0.14], *p* < 0.001) and linked to behavioral disengagement positively (β = 0.16, SE = 0.03, 95% CI = [0.10, 0.22], *p* < 0.001). In turn, both mediators were significantly associated with depressive symptoms at T3. Positive reframing at T2 negatively predicted depressive symptoms (β = −0.08, SE = 0.03, 95% CI = [−0.14, −0.02], *p* = 0.008), whereas behavioral disengagement at T2 positively predicted depressive symptoms (β = 0.14, SE = 0.02, 95% CI = [0.10, 0.18], *p* < 0.001). The indirect effect of perceived stress at T1 on depressive symptoms at T3 via positive reframing at T2 was significant (β = 0.018, SE = 0.006, 95% CI [0.006, 0.030], *p* = 0.003). Likewise, the indirect effect via behavioral disengagement at T2 was significant (β = 0.022, SE = 0.008, 95% CI [0.006, 0.038], *p* = 0.006). In addition, perceived stress at T1 had a significant direct effect on depressive symptoms at T3 (β = 0.20, SE = 0.04, 95% CI = [0.12, 0.28], *p* < 0.001), indicating partial mediation. A more detailed version of the longitudinal mediation model, including the autoregressive paths and covariate information, is presented in Supplementary Figure 1.

**Table 3:** Longitudinal mediation model.

Path	β	*SE*	95% CI	*p*
**Direct effect**	
Perceived stress at T1 → Depressive symptoms at T3	0.20	0.04	[0.12, 0.28]	< 0.001
**Indirect effect**	
Perceived stress at T1 → Positive reframing at T2 → Depressive symptoms at T3	0.018	0.006	[0.006, 0.030]	0.003
Perceived stress at T1 → Behavioral disengagement at T2 → Depressive symptoms at T3	0.022	0.008	[0.006, 0.038]	0.006

### 3.6 The moderated mediation

Stress mindset at T1 significantly moderated several pathways in the model. First, stress mindset moderated the association between perceived stress at T1 and positive reframing at T2 (β = 0.06, SE = 0.02, 95% CI = [0.02, 0.10], *p* = 0.003). Simple-slope analyses showed that the negative association between perceived stress and positive reframing was stronger at low stress mindset (β = −0.24) than at high stress mindset (β = −0.12). Second, stress mindset moderated the association between perceived stress at T1 and behavioral disengagement at T2 (β = −0.08, SE = 0.03, 95% CI = [−0.13, −0.03], *p* = 0.008). The positive association between perceived stress and behavioral disengagement was stronger at low stress mindset (β = 0.23) than at high stress mindset (β = 0.07). Third, stress mindset moderated the direct association between perceived stress at T1 and depressive symptoms at T3 (β = −0.08, SE = 0.03, 95% CI = [−0.13, −0.03], *p* = 0.004), with a stronger association at low stress mindset (β = 0.23) than at high stress mindset (β = 0.07). The results are shown in Table 4.

**Table 4:** Path coefficients for the moderated mediation model.

Path	β	*SE*	95% CI	*p*
Perceived stress at T1 → Positive reframing at T2	−0.18	0.04	(−0.26, −0.10)	< 0.001
Perceived stress at T1 ^*^ Stress mindset at T1 → Positive reframing at T2	0.06	0.02	(0.02, 0.10)	0.003
Perceived stress at T1 → Behavioral disengagement at T2	0.15	0.04	(0.07, 0.23)	< 0.001
Perceived stress at T1 ^*^ Stress mindset at T1 → Behavioral disengagement at T2	−0.08	0.03	(−0.13, −0.03)	0.008
Positive reframing at T2 → Depressive symptoms at T3	−0.10	0.03	(−0.16, −0.04)	0.001
Behavioral disengagement at T2 → Depressive symptoms at T3	0.08	0.02	(0.04, 0.12)	< 0.001
Perceived stress at T1 → Depressive symptoms at T3	0.15	0.04	(0.07, 0.23)	< 0.001
Perceived stress at T1 ^*^ Stress mindset at T1 → Depressive symptoms at T3	−0.08	0.03	(−0.13, −0.03)	0.004

Conditional indirect and direct effects at different levels of stress mindset are presented in Table 5. The indirect effect of perceived stress on depressive symptoms via positive reframing at T2 was significant at both low (β = 0.024, SE = 0.011, 95% CI [0.002, 0.046], *p* = 0.029) and high levels of stress mindset (β = 0.012, SE = 0.006, 95% CI [0.001, 0.024], *p* = 0.046), but the effect was larger at low stress mindset. The index of moderated mediation for this pathway was negative (β = −0.006), indicating that the indirect effect via positive reframing became weaker as stress mindset increased. Similarly, the indirect effect via behavioral disengagement at T2 was larger at low stress mindset (β = 0.018, SE = 0.006, 95% CI [0.006, 0.030], *p* = 0.003) than at high stress mindset (β = 0.006, SE = 0.003, 95% CI [0.001, 0.012], *p* = 0.046), with a negative index of moderated mediation (β = −0.006). The conditional direct effect of perceived stress on depressive symptoms was also stronger at low stress mindset (β = 0.230, SE = 0.011, 95% CI [0.208, 0.252], *p* < 0.001) than at high stress mindset (β = 0.070, SE = 0.014, 95% CI [0.043, 0.097], *p* < 0.001). The simple-slope plots for the significant interaction effects are presented in Supplementary Figure 2.

**Table 5:** Conditional indirect and direct effects at different levels of stress mindset at T1.

Effect	Low (−1 SD)			High (+1 SD)		
	*β (SE)*	95% CI	*p*	*β (SE)*	*95% CI*	*p*
Indirect via positive reframing at T2	0.024 (0.011)	[0.002, 0.046]	0.029	0.012 (0.006)	[0.001, 0.024]	0.046
Indirect via behavioral disengagement at T2	0.018 (0.006)	[0.006, 0.030]	0.003	0.006 (0.003)	[0.001, 0.012]	0.046
Direct effect	0.230 (0.011)	[0.208, 0.252]	< 0.001	0.070 (0.014)	[0.043, 0.097]	< 0.001

## 4 Discussion

The present study examined the longitudinal association between perceived stress and depressive symptoms among Chinese student-athletes, the mediating roles of positive reframing and behavioral disengagement, and the moderating role of stress mindset. Using a three-wave design, we found that higher perceived stress at T1 predicted greater depressive symptoms at T3, both directly and indirectly through lower positive reframing and higher behavioral disengagement at T2. In addition, these pathways were moderated by stress mindset, such that the associations of perceived stress were weaker among student-athletes with a stronger stress-is-enhancing mindset. By integrating longitudinal mediation and moderation within a single model, the present study extends existing stress-health research in athletic populations. It also provides a deeper understanding of how and under what conditions perceived stress is linked to depressive symptoms over time.

In the present study, student-athletes who reported higher levels of stress at T1 were more likely to report elevated depressive symptoms at T3, even after accounting for autoregressive effects. This finding is consistent with previous research conducted among students and athletic populations (Jochmann et al., 2024; Pänkäläinen et al., 2024). However, this study further strengthens the literature by showing that the association may develop over time. This temporal ordering is meaningful among student-athletes, whose daily lives are often characterized by sustained and overlapping demands (Ward et al., 2023). Many participants in our sample reported long training hours and extensive years of athletic involvement. Balancing intensive physical training and academic requirements may contribute to chronic strain, which makes them have limited opportunities to rest and recover (Jochmann et al., 2024; Pänkäläinen et al., 2024). Stress may gradually accumulate and become overwhelming under these conditions. Over time, these experiences erode emotional stability and increase vulnerability to depressive symptoms (Rice et al., 2016; Ward et al., 2023). In addition, the evaluative nature of competitive sport further intensifies stress appraisals. Repeated performance monitoring, selection pressures, and concerns about future athletic careers could heighten sensitivity to failure and threaten athletes' competence. These situations may help explain why perceived stress is closely linked to depressive symptoms in this population (Endo et al., 2023).

Our findings further showed that positive reframing and behavioral disengagement partially mediated the longitudinal association between perceived stress and depressive symptoms, supporting H1 and H2. More specifically, higher perceived stress at T1 predicted lower positive reframing at T2 and higher behavioral disengagement at T2, which in turn predicted higher depressive symptoms at T3. This result is in line with stress and coping theory (Folkman, 2013). Furthermore, the present findings point to two distinct mediation mechanisms through which stress is linked to depressive symptoms over time.

The first mediation (i.e., positive reframing) involves reduced constructive meaning-making. Positive reframing reflects the ability to reorganize adversity in a way that preserves meaning, manageability, and future orientation (Troy et al., 2018). This function is important among student-athletes. Many of the stressors, e.g., performance expectations, injury concerns, role conflict, and selection uncertainty, cannot always be removed immediately (Lau and Tov, 2023). In such contexts, psychological adjustment depends on whether individuals can reinterpret these demands as manageable, informative, or growth-promoting (Shiota and Levenson, 2012). However, when perceived stress is high, this ability easily becomes compromised. Individuals who feel overwhelmed become cognitively narrowed, emotionally exhausted, and less able to generate other interpretations of stressful experiences (Olsson et al., 2025). As a result, stress is experienced as threatening, which may be associated with hopelessness and negative self-evaluation, both of which are core features of depressive symptoms (Kara et al., 2025). Accordingly, lower use of positive reframing reflects greater difficulty in viewing adverse experiences from a more constructive perspective.

The second mediation involves progressive withdrawal from active engagement with stress. Behavioral disengagement signals the reduction in effort, persistence, and goal-directed coping when demands are appraised as exceeding one's resources (Monzani et al., 2015). As academic and athletic success rely heavily on sustained commitment, discipline, and repeated self-regulation, this mediation pathway is also important among student-athletes (Linnér et al., 2025). When student-athletes withdraw from effort under stress, they tend to avoid difficult tasks and give up after setbacks (Gulliver et al., 2012). This withdrawal gradually increases vulnerability to depressive symptoms (Yoon and Petrie, 2023). As problems remain unresolved and stress builds, they feel less in control and less capable of managing the situation (Xiang and Jiang, 2025). This is consistent with the positive pathway from behavioral disengagement to later depressive symptoms in the present study.

Notably, the two mediators should not be viewed as opposites. They capture two complementary processes in the development of depressive symptoms under stress. Lower positive reframing reflects a diminished capacity to cognitively transform the meaning of adversity (Lau and Tov, 2023), whereas higher behavioral disengagement reflects an increased tendency to withdraw effort from dealing with stressors (Monzani et al., 2015). The pathway involving positive reframing is cognitive and meaning-related, and the other (i.e., behavioral disengagement) is behavioral and action-related (Lau and Tov, 2023; Monzani et al., 2015). Including them simultaneously provides a more differentiated account of how perceived stress links to depressive symptoms. The present findings suggest that stress is associated with mental health by increasing maladaptive withdrawal and weakening adaptive meaning-focused coping. The distinction is important as interventions aimed at reducing depressive symptoms in student-athletes need to address both processes.

The present study further shows that stress mindset moderates the association of perceived stress with both coping pathways as well as on subsequent depressive symptoms, supporting H3 and H4. Specifically, among individuals with a stronger stress-is-enhancing mindset, perceived stress was less strongly associated with lower positive reframing and higher behavioral disengagement. The direct association between perceived stress and depressive symptoms was also weaker. These findings support the idea that stress mindset provides a broader framework for interpreting stressful experiences (Crum et al., 2013, 2017). Stress mindset shapes how individuals interpret the significance of stressors, even when the stressors remain unchanged. People who view stress as potentially beneficial may see demanding situations as challenges to work through, rather than as signs that they have failed (Kilby and Sherman, 2016). A stronger stress-is-enhancing mindset is associated with greater flexibility in interpreting stressful situations and continued engagement in coping. Constructive responses are therefore more likely, whereas withdrawal is less likely.

This moderating role of stress-mindset is meaningful among student-athletes. Stress is often embedded in daily routines of competition, evaluation, physical fatigue, and identity-relevant performance demands in this population. Sport culture generally socializes athletes to view pressure as an inevitable, and sometimes necessary part of progress and achievement (Endo et al., 2023; Yang et al., 2024). Student-athletes with a stronger stress-is-enhancing mindset tend to interpret pressure in ways that support continued effort, learning, and improvement (Crum et al., 2017). Such athletes are more able to sustain positive reframing even when demands are high (Kilby and Sherman, 2016). In contrast, when stress is primarily perceived as debilitating, the same demands will be read as threatening, exhausting, or uncontrollable, which may link to withdrawal-based responses (Jenkins et al., 2021). Stress mindset may affect how negatively individuals experience stress and whether they continue trying to manage the demands they face.

The moderation results suggest that stress mindset was associated with differences in the strength of these associations. Although the conditional indirect effects varied across levels of stress mindset, they remained significant at both low and high levels of the moderator. This indicates that a stress-is-enhancing mindset functions as a modest protective factor (Crum et al., 2017). It was associated with weaker adverse associations of perceived stress on coping and depressive symptoms. The interpretation is in line with prior research suggesting that beliefs about stress can shape emotional and physiological responses without fundamentally removing exposure to stress (Murphy et al., 2023; Park et al., 2018). The present findings point to a more detailed role of stress mindset. It may attenuate stress-related adverse associations and support adaptive functioning under demanding conditions (Murphy et al., 2023). Even student-athletes who endorse a relatively strong stress-is-enhancing mindset are still vulnerable to depressive symptoms when stress becomes chronic or when coping resources are not enough.

The magnitude of these effects should also be interpreted carefully. The indirect effects and moderation coefficients were statistically significant but modest in size. This pattern is common in longitudinal mediation models because prospective paths are estimated after controlling for autoregressive stability and relevant covariates, and indirect effects are calculated as the product of these residualized coefficients (Jose, 2016). Therefore, the present findings should not be interpreted as evidence of large clinical effects. Rather, they indicate modest longitudinal associations that may still be meaningful because stress, coping, and stress-related beliefs can operate repeatedly across training, academic, and competitive contexts.

Several alternative interpretations should also be considered. Although the three-wave design helped establish temporal ordering, the observed associations may still reflect reciprocal or dynamic processes. Student-athletes with higher depressive symptoms may appraise subsequent academic and athletic demands as more stressful and may have fewer psychological resources for positive reframing or sustained coping effort, which is consistent with recent longitudinal evidence suggesting that perceived stress and depressive symptoms may become interdependent over time (Samek and Dawson, 2024). Sport-type differences may also be relevant because student-athlete mental health is shaped by generic, sport-specific, and dual-career factors (Kegelaers et al., 2024), and athletes may experience different stressors depending on training, competition, evaluation, injury risk, and performance demands (Nuetzel, 2025). Although sport type was collected descriptively in the present study, the sample was not designed to test sport-specific moderation in a stable way. Cultural context may further shape the meaning of stress mindset among Chinese student-athletes, as recent evidence among Chinese students suggests that stress-related mindsets are associated with perceived-stress trajectories and later adjustment (Zhao et al., 2023). Future research should therefore examine reciprocal pathways, sport-specific stress contexts, and cultural factors when testing how perceived stress, coping strategies, stress mindset, and depressive symptoms are related over time.

The two mediation results have practical implications for supporting student-athletes' mental well-being, although these implications should be interpreted cautiously given the modest effect sizes. Interventions may benefit from efforts to address how athletes respond to stress alongside efforts to reduce stress exposure. Training in positive reframing and self-regulation may help student-athletes to stay engaged when facing difficulties. Coaches and mental health professionals should also identify withdrawal and loss of effort early, before these responses become established coping patterns (Nuetzel, 2023). This is relevant in the dual-career context, where academic and sporting demands often overlap and place additional pressure on student-athletes (Kegelaers et al., 2024). Recent research also suggests that mental health literacy, especially knowing where and how to seek appropriate support, is associated with better psychological outcomes among dual-career athletes (Usenik and Kranjec, 2025). The moderating results further suggest that student-athletes' beliefs about stress need attention in intervention programs. Recent sport-specific intervention research provides some support for this possibility. Mansell et al. (2024) found that a cognitive-behavioral program incorporating stress-mindset training improved stress mindset and reduced negative affect in young athletes, although it did not significantly improve anxiety or perceived performance. This finding also cautions against treating stress-mindset interventions as sufficient on their own. The moderation effects observed in the present study were modest, and changing beliefs about stress is unlikely to counter sustained high stress by itself. Such interventions may therefore be more appropriate when combined with coping-skills training and broader support for athletes facing persistent academic and sporting demands.

This study has several limitations. First, although the three-wave design strengthened temporal ordering, the observational design does not establish causality, and reciprocal pathways remain possible; for example, depressive symptoms may shape later stress appraisals and coping responses. Future experimental, intensive longitudinal, or cross-lagged studies should compare alternative specifications, including models controlling T1 depressive symptoms and reciprocal paths. Second, all measures were self-reported, which may introduce recall and reporting bias. Future research could include behavioral, observational, or physiological indicators. Third, positive reframing and behavioral disengagement were assessed with two-item Brief COPE subscales. Although reliability was acceptable, these observed scores provide limited construct coverage and may have lower measurement precision than longer coping measures. Future work should assess a broader coping repertoire and examine other mechanisms, such as social support, coaching climate, resilience, self-compassion, and sport-specific stressors. Fourth, participants were recruited from one Chinese province and from four institutions and 20 teams, which may limit generalizability and raise possible clustering concerns. Because anonymized institution, team, and coach identifiers were not retained, clustering could not be formally modeled. Future studies should retain anonymized cluster identifiers and recruit more institutions and teams. Finally, perceived stress was modeled as a single latent factor, whereas depressive symptoms and stress mindset were treated as observed scores. Future studies could test alternative PSS-10 specifications and latent models for depressive symptoms and stress mindset.

## 5 Conclusions

In conclusion, this three-wave longitudinal study showed that perceived stress was positively linked to later depressive symptoms among Chinese student-athletes. This longitudinal association was partially mediated by lower positive reframing and higher behavioral disengagement. Stress mindset further moderated these pathways, such that a stronger stress-is-enhancing mindset was associated with weaker links between perceived stress, coping, and depressive symptoms. Given the modest indirect and moderation effects and the reliance on self-report measures, these findings should be interpreted cautiously and do not establish the effectiveness of any intervention. By examining coping processes and stress mindset within the same longitudinal model, this study adds to the understanding of how perceived stress is associated with mental health among student-athletes managing the combined demands of academic study and athletic training.

## Data Availability

The raw data supporting the conclusions of this article will be made available by the authors, without undue reservation.
