# Supplementary material for: Longitudinal association between perceived stress and depressive symptoms among student-athletes: the mediating roles of positive reframing and behavioral disengagement and moderating role of stress mindset
Source: Front Psychol. 2026 Sep 8;17:1938720. doi: 10.3389/fpsyg.2026.1938720 (PMC13593483; doi:10.3389/fpsyg.2026.1938720)
Supplement: Supplementary file 1 [file Supplementary_file_1.docx]

Supplementary Figure 1. Detailed structural equation model of the longitudinal mediation pathways. Standardized coefficients are shown. Autoregressive paths were included for positive reframing, behavioral disengagement, and depressive symptoms. Age, sex, perceived family financial situation, years of training, and weekly training hours were included as covariates.

**p* < .05, ***p* < .01, ****p* < .001.

Supplementary Figure 2. Simple-slope plots of the moderating role of stress mindset. Panel A shows positive reframing, Panel B shows behavioral disengagement, and Panel C shows depressive symptoms. Values are plotted in standardized units. Low and high stress mindset represent -1 SD and +1 SD, respectively. The intercept was set to zero for visualization of the slope patterns.
