# Supplementary material for: Longitudinal association between perceived stress and depressive symptoms among student-athletes: the mediating roles of positive reframing and behavioral disengagement and moderating role of stress mindset
Source: Front Psychol. 2026 Sep 8;17:1938720. doi: 10.3389/fpsyg.2026.1938720 (PMC13593483; doi:10.3389/fpsyg.2026.1938720)
Supplement: Supplementary file 2 [file Supplementary_file_2.docx]

Supplementary Table 1 Attrition analyses

| Variable | Follow-up  (n = 769) | Lost to follow-up  (n = 71) | *p* |
| --- | --- | --- | --- |
| Categorical variable | n | % |  |
| Sex |  |  | 0.720 |
| Male | 493 (64.1) | 44 (62.0) |  |
| Female | 276 (35.9) | 27 (38.0) |  |
| Perceived family financial situation |  |  | 0.658 |
| Very poor/poor | 184 (23.9) | 18 (25.4) |  |
| Average | 539 (70.1) | 47 (66.2) |  |
| Good/very good | 46 (6.0) | 6 (8.5) |  |
| Type of sport |  |  | 0.729 |
| Team sports | 367 (47.7) | 31 (43.7) |  |
| Individual sports | 180 (23.4) | 17 (23.9) |  |
| Weight-class sports | 86 (11.2) | 11 (15.5) |  |
| Aesthetic sports | 136 (17.7) | 12 (16.9) |  |
| Weekly training hours |  |  | 0.244 |
| <5h | 75 (9.8) | 7 (9.9) |  |
| 5-10h | 61 (7.9) | 11 (15.5) |  |
| 11-15h | 152 (19.8) | 11 (15.5) |  |
| 16-20h | 239 (31.1) | 23 (32.4) |  |
| >20h | 242 (31.5) | 19 (26.8) |  |
| Continuous variable | Mean (SD) | Mean (SD) | *p* |
| Age | 20.3 (2.4) | 20.5 (1.3) | 0.484 |
| Years of training | 13.2 (2.1) | 13.6 (1.9) | 0.107 |
| Perceived stress | 14.8 (6.3) | 14.9 (6.2) | 0.919 |
| Stress mindset | 18.7 (4.3) | 18.3 (4.4) | 0.407 |
| Positive reframing | 6.2 (1.5) | 6.5 (1.4) | 0.062 |
| Behavioral disengagement | 3.12 (1.2) | 3.2 (1.3) | 0.741 |
| Depressive symptoms | 4.7 (3.6) | 4.6 (3.7) | 0.828 |

Supplementary Table 2 Factor loading and residual variance of PSS-10 items

| Item | Factor loading | Residual variance |
| --- | --- | --- |
| 1 | 0.64 | 0.60 |
| 2 | 0.60 | 0.64 |
| 3 | 0.61 | 0.63 |
| 4 | 0.60 | 0.64 |
| 5 | 0.60 | 0.64 |
| 6 | 0.60 | 0.64 |
| 7 | 0.51 | 0.74 |
| 8 | 0.62 | 0.62 |
| 9 | 0.55 | 0.70 |
| 10 | 0.66 | 0.57 |
